# P10 Haemophagocytic lymphohistiocytosis in the returning traveller with fever... especially if slow to defervesce on treatment!

**DOI:** 10.1093/rap/rkae117.041

**Published:** 2024-11-05

**Authors:** Sana Sharrack, Thomas Samuels, Sophie Skarbek, James Meiring, Danielle Cohen, Michael Brown, Jessica Manson, Rachel Tattersall

**Affiliations:** Department of Rheumatology, Sheffield NHS Foundation Trust, Sheffield, United Kingdom; Hospital for Tropical Diseases, Division of Infection, UCLH NHS Trust, London, United Kingdom; Division of Infection and Immunity, University College London, London, United Kingdom; Department of Infectious Diseases and Tropical Medicine, Sheffield Teaching Hospitals, Sheffield, United Kingdom; Department of Infectious Diseases and Tropical Medicine, Sheffield Teaching Hospitals, Sheffield, United Kingdom; School of Medicine and Population Health, University of Sheffield, Sheffield, United Kingdom; Department of Infectious Diseases and Tropical Medicine, Sheffield Teaching Hospitals, Sheffield, United Kingdom; Hospital for Tropical Diseases, Division of Infection, UCLH NHS Trust, London, United Kingdom; Clinical Research Department, London School of Hygiene & Tropical Medicine, London, United Kingdom; Department of Rheumatology, UCL NHS Trust, London, United Kingdom; Department of Rheumatology, Sheffield NHS Foundation Trust, Sheffield, United Kingdom

## Abstract

**Introduction:**

Haemophagocytic lymphohistiocytosis (HLH) is a potentially life-threatening hyperinflammatory disorder characterised by dysregulated immune activity resulting in malignant inflammation and multi-organ failure. Early recognition and treatment reduces mortality. HLH associated with rickettsial infection is described, and we present two cases of HLH secondary to severe rickettsial infection. We compare clinical and biochemical responses to IV methylprednisolone versus anakinra after HLH was suspected, identified and treated early in both presentations. We recommend a low index of suspicion for HLH for the returning traveller with fever where rickettsial infection is considered possible, especially if patients are slow to defervesce on treatment with doxycycline.

**Case description:**

Case 1 was of a 63-year old-male with no past medical history of note, who presented to hospital with fevers, rigors, loose stool and jaundice one week after returning from Ethiopia. He had stayed exclusively in Addis, had not visited any rural locations and did not recall any insect bites during his trip but had contact with one dog. On review, he denied any symptoms of systemic upset prior to his presentation and had no symptoms suggestive of an underlying rheumatological disease other than myalgia and arthralgia. On examination, he had no evidence of synovitis or rash, but had hepatosplenomegaly. He was diaphoretic with daily temperature spikes of up to > 39 degrees Celsius. He was investigated extensively as an inpatient, but no clear infectious trigger was identified. He was treated empirically on admission with IV ceftriaxone, but continued to deteriorate and a state of hyperinflammation was suspected. By day two of his admission, his HScore was 166 and he improved rapidly after treatment with oral doxycycline and two doses of IV methylprednisolone.

Case 2 was a 44-year-old male with no past medical history of note, who presented to hospital with fever, myalgia and arthralgia two weeks after returning from Cameroon. Again, he denied any rural travel and did not recall having sustained any insect bites or animal exposure. On review, he denied systemic upset prior to this presentation or other symptoms suggestive of an underlying rheumatological disease. Clinically, he had no rash, synovitis or organomegaly. He was also treated empirically with IV ceftriaxone. Two days later, initial infection screening was negative but fever persisted, and doxycycline was added. Fever persisted still, and by day four, HScore was 167. Anakinra was initiated, resulting in marked clinical improvement.

**Discussion:**

Fever in the returning traveller is often associated with serious illness and the initial focus of evaluation should be trying to identify infections that are potentially life-threatening, treatable or transmissible. In both cases, this was done and patients were investigated thoroughly and extensively, but no clear infectious trigger had been identified at the time of deterioration. Treatment was therefore empirical as both people were at risk of rickettsial infection. In both cases, systemic symptoms progressed and, despite a lack of immediate identification of a causal agent, there was recognition of a hyperinflammatory response to a likely infectious trigger. Hyperinflammation and HLH have a high mortality, prompting discussion in respective HLH MDTs for initiation of immunomodulation in parallel to anti-microbial treatment. Interestingly, in case one, doxycycline was started at the same time as IV methylprednisolone, which raises the question of whether the clinical and biochemical improvement was due to the antimicrobial treatment rather than corticosteroids. However in case two, there was no immediate improvement with introduction of oral doxycycline, and it was after anakinra was introduced at least two days later that there was a dramatic clinical and biochemical response.

Both cases raise the question of how much inflammation is acceptable in the context of acute inflammation when the body is trying to respond to, and tackle, an infectious agent. At what point does the inflammatory response become harmful, and is consideration and introduction of an immunomodulatory or immunosuppressive agent in the context of unidentified infection dangerous? What is the local pathway for urgent discussion of patients with hyperinflammation? These cases highlight that while the cause or trigger for HLH is unknown, the patient still needs immunomodulation promptly to prevent the potentially life-threatening manifestations of hyperinflammatory syndromes. Joint working between infectious disease and rheumatology is imperative.

**Key learning points:**

• It is recognised that systemic hyperinflammation and HLH can occur in nearly any inflammatory state, but certain predisposing conditions and/or triggers warrant a high index of suspicion. These cases suggest that rickettsial infection, when considered, should prompt the clinician to have a low index of suspicion for associated HLH, and that early recognition using the HScore and treatment can lead to a good outcome. Although the exact mechanisms that trigger HLH are not fully understood, it is interesting to consider why certain infections are more likely to cause it than others. In rickettsial disease, the fourth most common infectious group of diseases associated with HLH, it is likely that the cytokine storm associated with the immune response to rickettsial infection may be involved in the pathogenesis of complicated typhus that can lead to life-threatening complications such as ARDS, DIC and HLH. It is possible that early identification of HLH in our two cases with early control of the cytokine storm with immunosuppressive and immunomodulatory treatment halted progression of further life-threatening complications associated with rickettsial disease. Although treatment in our cases with doxycycline was started early, it is interesting to think what would have happened if treatment for identified HLH was started promptly, but specific anti-rickettsial treatment delayed. Would we have allowed the host more time to mount an appropriate level of response to infection pending decision about correct and definitive treatment, or would ongoing manifestations of life-threatening complications have ensued regardless? It is also interesting to note that in both cases, only a short course of immunosuppression was required to treat HLH which is not always the case with infectious triggers of HLH. This raises the question of whether early treatment can also lead to shorter duration of disease, and highlights the role of the MDT approach to HLH.

